# EPIRARE survey on activities and needs of rare disease registries in the European Union

**DOI:** 10.1186/1750-1172-7-S2-A22

**Published:** 2012-11-22

**Authors:** Domenica Taruscio, Sabina Gainotti, Luciano Vittozzi, Fabrizio Bianchi, Monica Ensini, Manuel Posada

**Affiliations:** 1 Italian National Centre for Rare Diseases, Istituto Superiore di Sanità, Roma, Italy; 2 National Council of Research, Pisa, Italy; 3 EURORDIS; 4 Istituto de Salud Carlos III, Madrid, Spain

## 

The EPIRARE project[1] aims to build consensus and synergies for the development of an EU platform for rare disease registries and to address relevant regulatory, ethical and technical issues associated with the registration of rare disease patients. To this aim, a survey was carried out among existing rare disease registries and databases to get information on their objectives, needs, governance mechanisms, sustainability, and measures for the compliance with regulatory and ethical requirements and for quality assurance, as well as expectations from and opinions on a registry platform. Responses were received from 255 registries, of which 220 active registries were selected based on the completeness of the response.

Among responding registries, 18, 61, 17 and 3% were international, national, regional or local. The fraction of registries population based, hospital based and following case series or cohorts was, respectively 56, 23 and 20%. Epidemiological and clinical researches were the most declared scopes (respectively 71, 61% in a multiple answer question) and characterized two clearly different clusters of registries. Treatment efficacy and safety was the scope indicated by 45% registries. A wide heterogeneity is found regarding the disease coding system used, with 62% using their own or no code. Twenty-seven percent registries are established by law or to comply with regulatory requirements, while 73% as part of research projects or as an autonomous decision of clinicians or patients. Half of the registries shares data with other registries and 33% with centres of expertise, while 30% do not exchange with neither of them or with bio banks. Registries were established with no initial funding (21%) or with funding by public authorities (37%), industries and foundations (21%), research institutes and hospitals (25%), patients associations (16%) and the European Commission (15%). After the initial phase, more registries (25%) are run without specific funding; frequency of funding sources remains stable but for the European Commission, which decreases to 6%. Different procedures are applied for quality assessment, but each of them is applied by 46-58% registries. A main governing body is not present in 34% registries and 48% registries have no policy to ensure long-term sustainability. Main needs expressed are financial support, improved communication strategies and more extended geographical coverage, data sources and registry networking. The vast majority of respondents (80%) is favourable to a platform for registries and 60% doubts that new legislation can facilitate registration. Popularly expected platform services are technological tools, specific expert advice and resources.
